# Simultaneous 2D Strain Sensing Using Polymer Planar Bragg Gratings

**DOI:** 10.3390/s150204264

**Published:** 2015-02-12

**Authors:** Manuel Rosenberger, Waltraud Eisenbeil, Bernhard Schmauss, Ralf Hellmann

**Affiliations:** 1 Applied Laser and Photonics Group, University of Applied Sciences Aschaffenburg, Wuerzburger Strasse 45, 63743 Aschaffenburg, Germany; E-Mails: s8448@h-ab.de (W.E.); ralf.hellmann@h-ab.de (R.H.); 2 Institute of Microwaves and Photonics, University of Erlangen-Nuernberg, Cauerstrasse 9, 91058 Erlangen, Germany; E-Mail: bernhard.schmauss@fau.de

**Keywords:** optical sensing, planar Bragg grating, polymers, tensile and compressive strain, single writing step

## Abstract

We demonstrate the application of polymer planar Bragg gratings for multi-axial strain sensing and particularly highlight simultaneous 2D strain measurement. A polymer planar Bragg grating (PPBG) fabricated with a single writing step in bulk polymethylmethacrylate is used for measuring both tensile and compressive strain at various angles. It is shown that the sensitivity of the PPBG strongly depends on the angle between the optical waveguide into which the grating is inscribed and the direction along which the mechanical load is applied. Additionally, a 2D PPBG fabricated by writing two Bragg gratings angularly displaced from each other into a single polymer platelet is bonded to a stainless steel plate. The two reflected wavelengths exhibit different sensitivities while tested toward tensile and compressive strain. These characteristics make 2D PPBG suitable for measuring multi-axial tensile and compressive strain.

## 1. Introduction

Since the development of the first optical Bragg gratings by Hill *et al.* [1] they have been under constant investigation in a wide range of applications such as, e.g., components in optical networks, lasers and optical sensor elements. Starting with Bragg gratings in silica fibers [2–4], to planar Bragg gratings in silica based multi-layer systems [5–7], nowadays polymer materials especially attract increasing interest for the realization of Bragg grating sensors [8–10]. Due to their cost-effectiveness, biocompatibility, distinct material properties, and their photosensitivity polymers are advantageous in multiple applications [11,12]. It has already been demonstrated that Bragg gratings in polymer optical fibers can be used for bio-, humidity and temperature sensing as well as for measuring tensile strain [13–16]. Especially for the latter, polymers are as compared to silica well suitable materials due to their lower Young's modulus and their higher breaking elongation. In recent years, mainly two different kinds of polymer materials, namely TOPAS, a cyclo-olefin copolymer, and polymethylmethacrylate (PMMA), are under investigation in the field of strain sensing with polymer optical fiber Bragg gratings [17–19]. Multiple studies revealed that polymer optical fiber Bragg gratings show a very sensitive behavior towards tensile strain as well as a low hysteresis if properly mounted [20,21]. Furthermore, it has been shown by the authors that contrary to fiber-based Bragg grating sensors the application of polymer planar Bragg gratings (PPBG) is not restricted to measure tensile but also compressive strain without any further modification [22–24]. However, as yet only the properties of PPBG related to strain alongside the optical waveguide including the grating have been evaluated. In contrast to that, from an application point of view it is highly desirable to facilitate multi-axial strain measurements to record real mechanical load cases, which not necessarily coincide with the direction of a single fiber Bragg grating.

In this work, we demonstrate the ability to measure multi-axial strain with a polymer planar Bragg grating sensor. Firstly, the behavior of a PPBG at different angles between the waveguide including the Bragg grating and the direction of the mechanical load is evaluated. Starting with applying strain alongside the optical waveguide, *i.e.*, the angle is zero degrees, tensile strain results in a spectral red shift of the reflected Bragg wavelength while compressive strain leads to a comparable spectral blue shift. For an increasing angle between the waveguide and the direction of the mechanical load, *i.e.*, if the PPBG is rotated about the direction of the load, the PPBG based strain sensor exhibits a reduced sensitivity until there is almost no effect of the mechanical load on the Bragg wavelength at an angle of 45°. Upon further rotation, the reflected Bragg wavelength reveals a reversed behavior, *i.e.*, it is decreasing with tensile and increasing with compressive strain until the sensitivity reaches a maximum at 90°. Based on these results, a two dimensional polymer planar Bragg grating sensor is fabricated by writing two Bragg gratings with an angular offset into a single PMMA substrate. The gratings are overlapping in the middle of the PMMA platelet and each of the waveguide structures is butt coupled and glued to a single mode fiber providing two independent Bragg reflections as sensing signals. After bonding the 2D PPBG to a metal substrate tensile and compressive strain measurements reveal the directional dependence of the 2D PPBG and its applicability in multi-axial strain sensing.

## 2. Experimental Section: Sensor Fabrication

Polymer planar Bragg gratings (PPBGs) are fabricated in commercially available bulk polymethylmethacrylate (PMMA) with a thickness of 550 μm and a lateral length of 22.9 mm (Goodfellow GmbH, Bad Nauheim, Germany) which is cut to size with a precision saw (Secotom 15, Struers GmbH, Willich, Germany) and the cutting edges being polished with an advanced preparation system (Tegramin 20, Struers GmbH). Both Bragg grating and optical waveguide are fabricated in a single writing step by aligning an amplitude mask with a 15 μm wide waveguide structure above a phase mask exhibiting a grating period of 1053 nm. The stacked mask configuration is placed in contact with the prepared PMMA platelets and illuminated with a KrF excimer laser (Coherent Bragg Star, Coherent GmbH, Dieburg, Germany) emitting at a wavelength of 248 nm. A detailed description of the rapid fabrication process of a polymer planar Bragg grating in bulk PMMA is given in Ref. [25]. Please note that for the 2D PPBG the round PMMA substrate with a thickness of 1.1 mm and a diameter of 22.8 mm is cut with a CO_2_ laser and an additional second phase mask with a grating period of 1036.79 nm is used. This facilitates the simultaneous detection of the PPBG sensor response in two directions at distinct spectral positions.

After butt coupling a single mode fiber (SMF) to the optical waveguide and bonding it using UV curable adhesive, the PPBG can be connected to standard optical interrogation equipment. The measurement setup which is used for recording the reflected Bragg wavelength exhibits a sampling rate of 2 Hz and a resolution of 1 pm. It consists of a tunable laser diode emitting a wavelength spectrum from 1510 nm to 1590 nm, a circulator, and a photo diode (MicronOptics Inc, Atlanta, GA, USA).

To characterize the response of the PPBG towards tensile and compressive strain at different angles, a universal tester (Shimadzu GmbH, Duisburg, Germany) with custom made clamping tools is used. During the automated testing scheme the applied force as well as the travelled distance is recorded with a sampling rate of 100 Hz.

**Figure 1.**
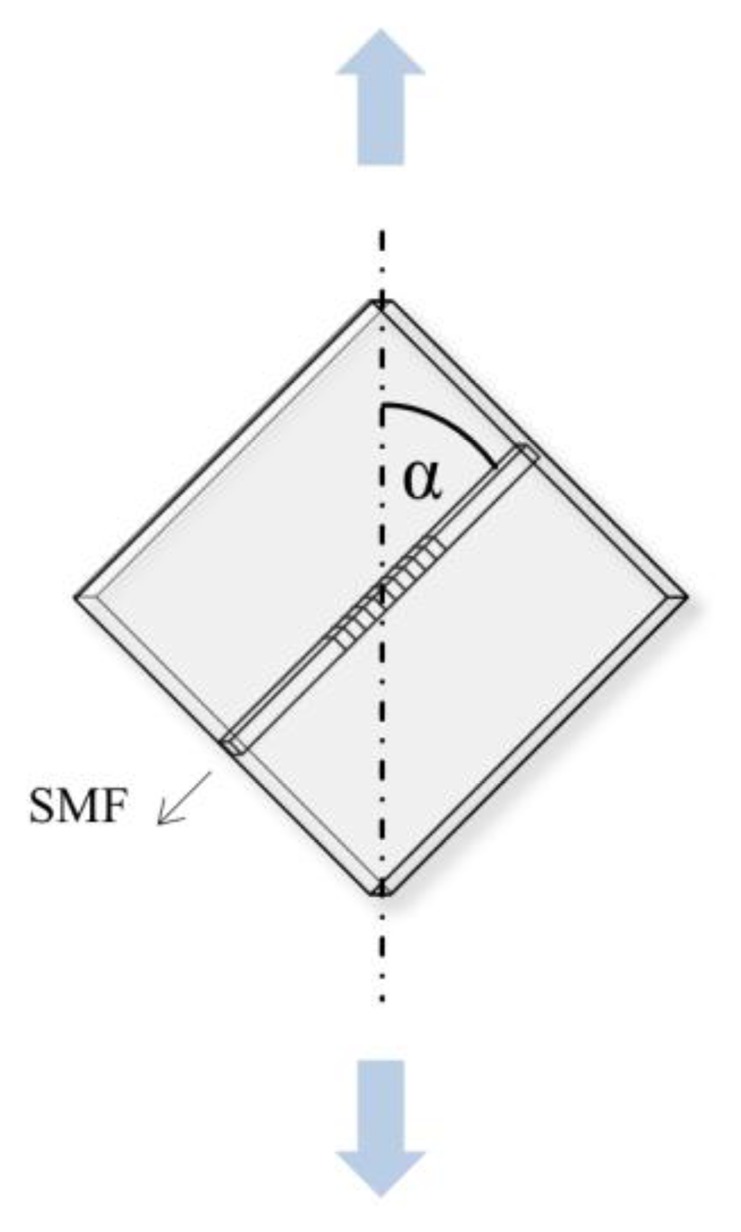
Orientation of the PPBG sensor with respect to the direction of load transmission (indicated by blue arrows).

The polymer planar Bragg grating sensor is loaded with tensile and compressive strain under different angles between the waveguide and the direction of the load transmission. Starting from an unloaded state the PPBG is lengthened by 3 μm, subsequently unloaded before being compressed by −3 μm. Following, the magnitude of the mechanical load is increased to ±5 μm, ±8 μm, ±10 μm, ±13 μm and ±15 μm, respectively. The described testing scheme is applied to the PPBG at different angles (α), namely 0°, 30°, 45°, 60° and 90°. A schematic illustration of the PPBG and the applied force at these angles is given in Figure 1.

**Figure 2.**
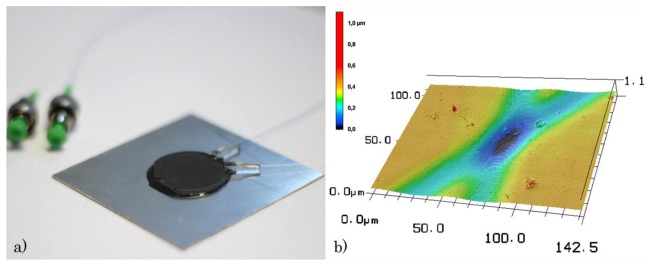
(**a**) Illustration of the 2D PPBG sensor chip in PMMA ontop a metal substrate. The two planar Bragg gratings intersect in the center of the PMMA platelet; (**b**) laser scanning microscopy image of the sensor chip surface at the intersection of the two waveguides with Bragg grating having an angular offset of 40°.

In addition, the second sensor with two gratings overlapping in the center of the round platelet at an angular offset of 40° is fabricated with the same writing technique in two consecutive writing steps. Each writing step generates the waveguide and the Bragg grating in one direction. For concurrent measurement of the 2D PPBG sensor response, two phase masks exhibiting different grating periods of Λ_1_ = 1036.79 nm and Λ_2_ = 1053 nm, respectively, are employed. As a result, the gratings in each direction display Bragg reflections at distinct spectral positions. Each waveguide including an integrated Bragg grating is butt coupled to a single mode fiber and subsequently bonded to a stainless steel plate facilitating simultaneous detection of the reflected Bragg wavelength of both gratings. The configuration of the multi-axial strain sensor including the two SMFs ontop of the square stainless steel plate (lateral length: 55 mm, thickness: 0.6 mm) is illustrated in Figure 2a.

The intersection of the two Bragg gratings is recorded with a laser scanning microscope (Keyence, Deutschland GmbH, Neu-Isenburg, Germany) as depicted in Figure 2b. Apparently, the intersection area exhibits a higher degree of degradation as compared to the individual waveguide regions. This is assigned to the twofold UV illumination of the intersection area during the writing steps of each individual waveguide. Nevertheless, the reflected spectra of both Bragg gratings exhibit the same characteristics (spectral position, reflected intensity, FWHM) as compared to previously reported PPBGs after a single exposure [23]. This can be attributed to the fact that the selected parameters of the UV writing process (fluence = 8 mJ/cm^2^, laser repetition rate = 200 Hz, number of pulses = 3000) result in volume gratings with an effectual distance to the surface, as shown in [25]. As a consequence, the Bragg gratings remain unaffected by the slight surface roughening resulting from the twofold UV illumination.

The 2D polymer planar Bragg grating is strained by applying forces of ±30 N, ±50 N, ±80 N and ±100 N onto the steel plate, respectively. It is worthwhile to mention that at 0° the force is applied alongside one planar Bragg grating and with an angular offset (40°) to the second Bragg grating. Following, the sensing device is rotated and loaded at 45° and 90° with respect to Λ_1_. By inserting a 2 × 2 single mode coupler with a split ratio of 50:50 between the 2D PPBG and the Bragg meter the reflected Bragg wavelengths of both gratings are recorded simultaneously during these measurements.

## 3. Results and Discussion

**Figure 3.**
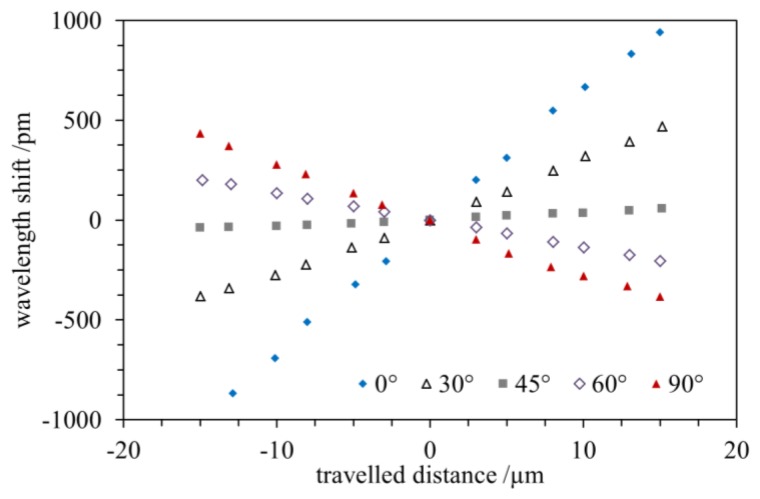
Spectral shift of the Bragg wavelength *vs.* travel distance for different orientations between the waveguide and the direction of the load transmission. The sensitivity of the PPBG sensor is highest for parallel orientation (0°) and almost vanishes at an angle of 45°.

The spectral shift of the Bragg wavelength upon the application of a mechanical load according to the above specified testing scheme is shown in Figure 3 for different angles between the orientation of the waveguide and the direction of the load transmission. For these measurements the PPBG sensor is clamped according to Figure 1 and the angle has been set to 0°, 30°, 45°, 60° and 90°, respectively. A positive travel distance corresponds to tensile strain, whereas a negative travel distance relates to compressive strain.

In accordance to previous findings, for parallel orientation at an angle of 0° between the waveguide and the direction of the mechanical load transmission the Bragg wavelength of the PPBG sensor exhibits a spectral shift towards longer wavelengths upon tensile strain and a spectral shift towards shorter wavelengths upon compressive strain [23]. In the investigated range of tensile and compressive strain, the Bragg wavelength exhibits a linear dependence on strain. While a linear dependence is also observed at an angle of 30°, the sensitivity (wavelength shift over travelled distance) of the PPBG sensor is reduced. Most strikingly, at an angle of 45° the PPBG sensor reveals almost no response upon compressive or tensile strain. Rotating the PPBG sensor further to 60° results in an opposite linear behavior, *i.e.*, tensile strain leads to a spectral blue shift and accordingly compressive strain to a spectral red shift of the Bragg wavelength. A maximum negative wavelength shift upon tensile strain is obtained at an angle of 90°.

**Figure 4.**
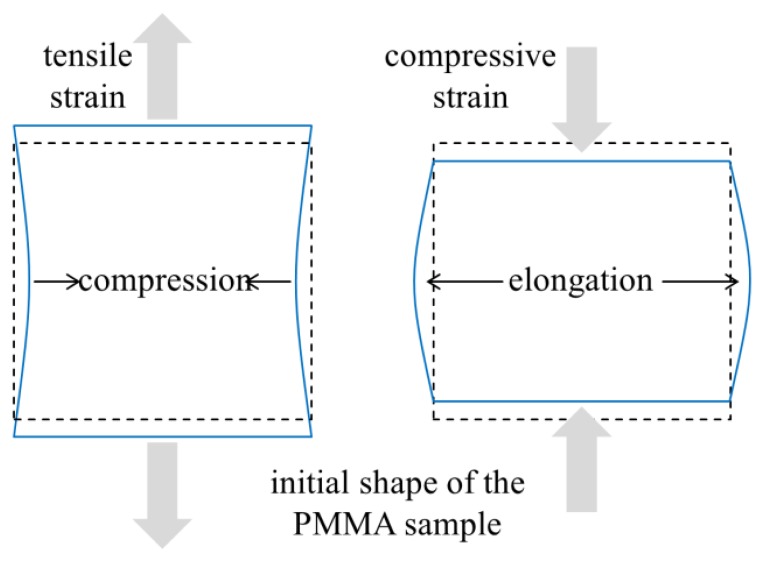
Schematic illustration of the deformation of the PMMA sample caused by tensile strain (**Left**) and compressive strain (**Right**).

This behavior of the PPBG sensor can be explained by the deformation of the PMMA sample upon tensile and compressive strain. Figure 4 depicts a schematic illustration of the sensor chip during the measurement. It is evident from Figure 4 that the applied tensile strain lengthens the vertical axis of the PMMA platelet PPBG and concurrently compresses the horizontal axis. This results in a longer Bragg grating period at an angle of 0°. In contrast, it results in a shorter Bragg grating period at an angle of 90°. On the other hand, compressive strain shortens the vertical axis and lengthens the horizontal axis, leading to a reversionary alternation of the Bragg grating period. Since the reflected Bragg wavelength λ*_B_* is related to the grating period Λ*_B_* by:

(1) $$m\cdot \lambda_{B}=2\cdot n_{\text{eff}}\cdot \Lambda_{B}$$

with *m* being the order of the Bragg grating and *n_eff_* denoting the effective refractive index, λ*_B_* varies linearly with the grating period [2]. As a consequence, changes of the grating period result in a spectral shift of the reflected Bragg wavelength. The observed opposing behavior of the reflected Bragg wavelength at different angles, as shown in Figure 3, is caused by a contraction and bowing of the planar PMMA sensor chip during tensile and compressive strain. Accordingly, for an orientation of 45° the PPBG shows almost no response to tensile or compressive strain as the induced changes in length and width compensate each other.

**Figure 5.**
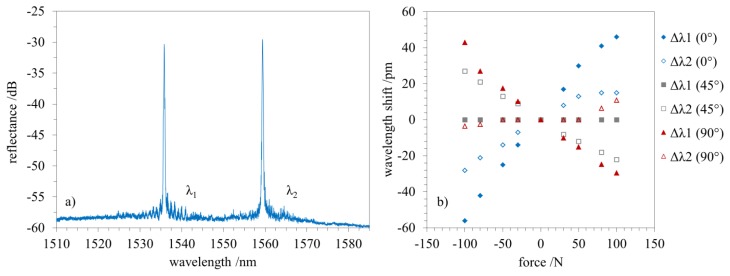
Multi-axial strain sensing using a 2D PPBG. The two angular displaced planar Bragg gratings exhibit an unequal spectral response towards tensile and compressive strain. (**a**) reflected spectrum recorded using a 2 × 2 50/50 coupler; (**b**) sensitivity towards tensile and compressive strain at various angles.

By using a 2D PPBG as shown in Figure 2, we further on demonstrate the applicability of a single polymer sensor chip to simultaneously measure multi-axial strain. The results depicted in Figure 5 reveal the spectral characteristics (Figure 5a) and the sensing behavior of the two planar Bragg gratings during tensile and compressive strain.

Figure 5b displays the sensitivity of the reflected Bragg wavelengths for the two gratings at different angles, *i.e.*, 0°, 45°, 90° with respect to Λ_1_. The y-axis represents the wavelength shift while the x-axis shows the applied force of the universal tester (positive values correspond to tensile strain, and negative values correspond to compressive strain).

The reflected wavelength of the PPBG (Λ_1_) which is oriented alongside the direction of the force (0°) reveals a spectral red shift while being strained and a corresponding spectral blue shift during compression while the angular shifted (40°) PPBG (Λ_2_) exhibits similar behavior with a reduced sensitivity. Please note, that the deviation from the linear behavior depicted in Figure 5b is attributed to the bonding of the 2D PPBG to the metal substrate since the strain measurements depicted in Figure 3 reveal a linear sensitivity of the PPBG towards tensile and compressive strain at different angles. To stress the angle-dependent sensitivity of the two Bragg gratings the 2D PPBG is rotated by 45° and 90°. Whereas, λ_1_ shows no reaction while loaded at 45°, λ_2_ exhibits a blue shift during tensile and a red shift during compressive strain. Further rotation to 90° demonstrates an inverse behavior of λ_1_ compared to 0°, which is in good correspondence to the results already described in Figure 3. Based on the wavelength shifts and their specific ratio, the 2D PPBG can be used to determine the applied force and the associated angle if the type of strain (tensile or compressive) is known.

## 4. Conclusions

In summary, we have described the behavior of a polymer planar Bragg grating towards tensile and compressive strain under various angles between the orientation of the planar grating and the direction of the mechanical load transmission. For strain applied alongside the grating, here denoted as 0°, the PPBG strain sensor exhibits the highest sensitivity against tensile strain revealing a spectral red shift of the Bragg wavelength while compressive strain leads to a spectral blue shift. With increasing angle between the grating orientation and the applied strain, the sensitivity decreases until for an angle of 45° the PPBG sensor shows an almost negligible wavelength shift. By further rotating the PPBG sensor, tensile strain results oppositely in a spectral blue shift and compressive strain in a spectral red shift with the highest sensitivity accomplished at an angle of 90°.

Furthermore, we have demonstrated the application of a 2D polymer planar Bragg grating structure for simultaneous multi-axial tensile and compressive strain sensing. The 2D PPBG consists of two Bragg gratings written into a single planar PMMA substrate angularly shifted by 40°. Upon mechanical load with varying strain the grating oriented parallel to the load exhibits the typical signature of a spectral red shift upon tensile strain and a spectral blue shift upon compressive strain. Most strikingly, the simultaneously detected spectral response of the second grating displays a proportionately displaced behavior according to the angular offset. In conjunction with the rapid fabrication process of the sensor structure, the sensitivity characteristic, the 2D‐PPBG offers a suitable device approach for polymer optical multi-axial strain sensing.
